# Functional Genomics in Pancreatic β Cells: Recent Advances in Gene Deletion and Genome Editing Technologies for Diabetes Research

**DOI:** 10.3389/fendo.2020.576632

**Published:** 2020-10-08

**Authors:** Ming Hu, Ines Cherkaoui, Shivani Misra, Guy A. Rutter

**Affiliations:** ^1^Section of Cell Biology and Functional Genomics, Faculty of Medicine, Imperial College London, London, United Kingdom; ^2^Metabolic Medicine, Department of Metabolism, Digestion and Reproduction, Faculty of Medicine, Imperial College London, London, United Kingdom

**Keywords:** genome editing, beta cell, genome-wide association studies, maturity onset of diabetes of the young, stem cells, mouse models

## Abstract

The inheritance of variants that lead to coding changes in, or the mis-expression of, genes critical to pancreatic beta cell function can lead to alterations in insulin secretion and increase the risk of both type 1 and type 2 diabetes. Recently developed clustered regularly interspaced short palindromic repeats (CRISPR/Cas9) gene editing tools provide a powerful means of understanding the impact of identified variants on cell function, growth, and survival and might ultimately provide a means, most likely after the transplantation of genetically “corrected” cells, of treating the disease. Here, we review some of the disease-associated genes and variants whose roles have been probed up to now. Next, we survey recent exciting developments in CRISPR/Cas9 technology and their possible exploitation for β cell functional genomics. Finally, we will provide a perspective as to how CRISPR/Cas9 technology may find clinical application in patients with diabetes.

## Introduction

Type 2 diabetes (T2D) affects an estimated 425 million people worldwide, a number predicted to rise to 629 million by 2045 (1). The disease usually involves insulin resistance but is ultimately the result of pancreatic β cell failure, a *sine qua non* for disease development (2). In contrast, Type 1 diabetes (T1D) affects a smaller proportion of people with diabetes and is chiefly the result of pancreatic β cell destruction mediated by immune cells (3).

Both genetic susceptibility and environmental drivers, notably obesity and sedentary lifestyles, determine the overall risk of T2D (4–6). Supporting a genetic component, rare monogenic forms of the disease exist with Mendelian inheritance (7, 8). Thus, maturity onset of diabetes of the young (MODY) is a rare form of diabetes with mutations often residing in exons encoding the functional domains of transcription factors such as hepatocyte nuclear factor hepatocyte nuclear factor 1 homeobox A (HNF1A) (9) and HNF4A (10), or of proteins involved in β cell glucose metabolism such as glucokinase (*GCK*) (11) (**Table 1**).

**Table 1:** Details of MODY genes.

MODY Gene	Gene Function	Related Disease/Phenotype	Ref
HNF4A	Transcription factor	Progressive β cell dysfunction Neonatal Hyperinsulinemic Hypoglycemia (HH) or diazoxide-responsive HH Sensitivity to sulphonylureas Macrosomia	(10)
GCK	Enzyme in the first step of glucose metabolism	Progressive β cell dysfunction Hyperglycaemia Reduced insulin secretion Reduced hepatic glycogen synthesis and stores	(11)
HNF1A	Transcription factor	Progressive β cell dysfunction Reduced β cell proliferation and increased apoptosis Glycosuria, sensitivity to sulphonylureas High concentration of High-Density Lipoprotein (HDL) cholesterol	(9)
PDX1	Transcription factor	Neonatal diabetes, Pancreatic developmental anomalies	(12)
HNF1B	Transcription factor	β cell dysfunction and insulin resistance Syndrome of Renal Cysts and Diabetes (RCAD) Hyperuricemia, abnormal liver function tests and hypomagnesaemia	(10)
NEUROD1	Transcription factor	β cell dysfunction Syndrome of permanent neonatal diabetes and neurological abnormalities	(13, 14)
CEL	Controls exocrine and endocrine functions of pancreas	Faecal elastase deficiency and pancreatic exocrine dysfunction Fat malabsorption	(15)
INS	Encode the proinsulin precursor	Permanent Neonatal Diabetes MODY (PNDM)	(16)
ABCC8	Regulating insulin release	Neonatal diabetes Congenital hypoglycemia hyperinsulinism (CHI)	(17)
KCNJ11	Regulating insulin release	Neonatal diabetes CHI	(18)
APPL1	Insulin signal pathway	Insulin-response defect: insulin action and secretion	(19)
RFX6	Transcription factor	Directing islet formation and insulin production	(20)
GATA6	Transcription factor	Neonatal diabetes Complete absence of the pancreas or an extreme reduction in its size	(21, 22)
PTF1A	Transcription factor	Neonatal diabetes Complete absence of the pancreas	(23)
EIF2AK3	Protein synthesis	Modulating the trafficking and quality control of proinsulin	(24, 25)

In most cases, however, T2D is a complex polygenic trait and the search for disease-associated variants has been underway for more than three decades. Genome-wide association studies (GWAS) (6, 26–31) have now identified >500 *loci* in the human genome which alter T2D risk. The majority of the identified variants affect insulin secretion from pancreatic β cells, rather than insulin action (29). Similar to other complex diseases (32), identified genetic variants confer relatively small increments in risk for T2D and explain only a small proportion of heritability (6). Such “missing heritability” (32) raises many questions, including whether a person’s susceptibility to disease may depend more on the combined effect of all the variants in the “background” than on the disease variants in the “foreground”. In any case, the impact of the risk variants may depend on genetic context (including modifier genes). This situation is further complicated by disease heterogeneity, with four sub-classes of T2D recently being defined by categorical K-means clustering (33) [but see (34) for an alternative description of heterogeneity]. We note that the above interactions complicate the assessment of risk heritability at the population level, such that an overestimate cannot be ruled out.

GWAS indicates that multiple genes and pathways are likely to be involved in disease development, consistent with the very large number of variants now associated with disease risk. Indeed, interactions between tissues may mean that effects, for example on insulin secretion, may, in fact, reside not at the level of the pancreatic β cell, but rather (at least in part) in other tissues from which regulatory molecules are released, for example adipokines secreted by fat cells, which then go on to influence β cell function (35). Nevertheless, as an initial step, it is reasonable to focus on the identified variants, and the likeliest site of action (the pancreatic β cell in the case of T2D) with the goal of elucidating their impacts at the molecular and cellular level, and consequently on disease pathogenesis. More sophisticated studies, exploring inter-organ communication, for example through cell-type selective inactivation in the “non-canonical” tissue in animal models (35), or in extra-pancreatic cells types, may nonetheless be warranted to achieve an in-depth understanding of the full spectrum of actions of a given variant. Clearly, though, the long list of gene variants and of disease-relevant issues make the number of testable combinations huge and, in our view, it will be important to design experiments carefully to test targeted hypotheses.

The identified genetic variants can be divided into two categories: those in protein-coding regions (fewer than 10%) and those (> 90%) in non-coding (intergenic and intronic) regions (36, 37). Variants in protein-coding regions create changes in amino acid sequence and, as a result, may impair protein function or stability. Protein truncation and loss of binding capacity to DNA or an interaction domain with other proteins are commonly observed in transcription factors (38). These variants are therefore obvious targets for mechanistic studies, are potentially targets for drug therapy, and have been the subject of several recent studies (5, 6). We note that rare coding variants of genes at *loci* hosting common variants appear to contribute only ~ 25% of overall T2D risk (39).

How do intragenic or intergenic variants alter cellular function or viability, and hence impact pathogenesis? Epigenetic studies of human islets have yielded a huge amount of information on where gene regulatory elements are located on chromosomes. For example, transposase-accessible chromatin sequencing (ATAC-seq) analysis (40) identifies open chromatin regions whilst Chromatin immunoprecipitation sequencing (CHIP-seq) assays on histones and transcription factors identifies histone modifications associated with active transcription or with transcriptional repression. These approaches have identified enhancer regions and enhancer clusters characterized by the acetylation of histone H3 lysine 27 (H3K27ac) (41, 42). Enhancer clusters, also termed stretch enhancers (41) or super-enhancers (43, 44), are enriched for gene regulatory elements and can be highly islet specific. Combined with GWAS data it has been possible to show that T2D variants are enriched in islet-specific enhancer clusters (41, 42).

Importantly, studies of the 3D structure of chromatin using chromatin conformation capture (3C) and 3C-based techniques (45, 46) can reveal the physical relationship between an enhancer cluster and its target genes (47, 48). Enhancer clusters often regulate multiple genes through loop formation (48). Causal variants therefore usually reside in the active enhancer region, from where they may influence enhancer activity and thus the expression of multiple target genes.

There are several challenges in not only interpreting the information above but ultimately translating this into benefit for those living with T2D. Firstly, how can we determine which of the multiple variants that are often found at a given locus, and may be co-inherited (i.e. in strong linkage disequilibrium), is responsible for altering disease risk? Secondly, through which downstream gene(s) do these act? Thirdly, how do changes in the expression of these genes affect cellular physiology? Which cell types and systems are involved? Whilst pancreatic β cells are the most likely to be affected where insulin secretion is changed, actions of variants in other tissues, which are either sensitive to insulin or may lie “upstream” of β cells in regulatory circuits that control insulin output (e.g. entero-endocrine cells or neurones) (35), may also be involved.

**Figure 1:**
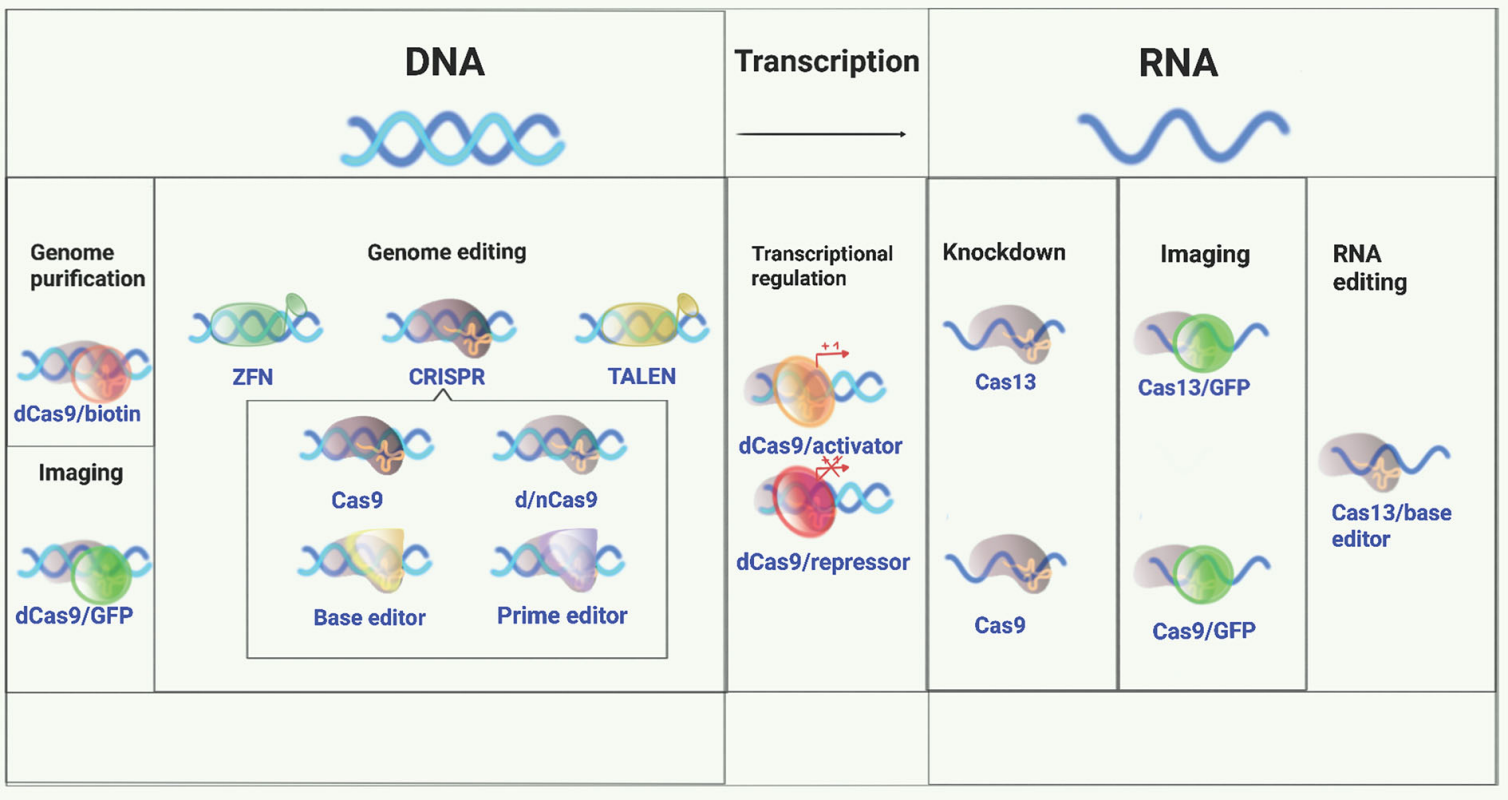
A versatile genome-editing toolbox. Following the original demonstration of genome editing, applications of ZFN, TALEN, and CRISPR for genome editing, regulation, monitoring, and beyond were subsequently developed (49). Conceptually, there are three major application tracks: 1. genome editing, including gene knockout, knockin, and indel formation initiated with a double strand DNA break made by a nuclease; 2. gene regulation and delivery of various functional moieties (e.g., transcription factors) to unique sites in DNA using catalytically inactivated derivatives of the same nucleases; 3. targeting single strand RNA for inactivation, editing, modification, or localization.

Genome editing tools that target the desired genomic region and allow for variants to be altered (e.g. from risk to protective), or for more substantial changes to be made (e.g. the deletion of a longer stretch of DNA harbouring a number of variants) and can help to answer each of these questions. These technologies are evolving rapidly (**Figure 1** and **Table 2**). The most recently developed of these, Clustered Regularly Interspaced Short Palindromic Repeat (CRISPR) technology, originally developed by Doudna, Charpentier and their colleagues (72, 73) and Zhang and his colleagues (50) has become a widely used tool for this purpose. Engineered CRISPR/Cas9 technology uses a guide RNA (gRNA) to direct CRISPR-associated endonuclease (Cas) to the target DNA and generate a double strand DNA break. Correction of a mutation or variant in the target DNA sequence can then be carried out by homology-directed DNA repair (HDR) with a donor template. Since its discovery eight years ago, CRISPR technology has evolved quickly to be a critical part of the molecular biologist’s toolbox.

**Table 2:** Applications of gene/genome editing tools.

FUNCTION	TYPE of CRISPR	APPLICATION	REF
**DNA**			
Double strand DNA break and indel formation	Cas9 Cas12a (Cpf1) Cas12e (CasX)	Gene knockout; DNA deletion; Knock-in by HDR	(50–52)
Single strand DNA break	nCas9 (Nickase)	Gene Knockout, DNA deletion.	(53)
Imaging of genomic DNA	dCas9-Suntag dCas9Rainbow dCas9-sirius	Visualization of genomic DNA locus under fluorescent microscope	(54–56)
Genomic DNA purification	dCas9-Flag dCas9-Biotin	Chromatin immunoprecipitation by antibody against tag protein or Cas9 protein.	(57, 58)
Genome screen	gRNA library	Identification of genes or genetic loci in cellular function	(59)
Base editing	nCas9-APOBEC nCas9-ABE nCas9-AID	Cytidine deaminase: converting C to U to T Adenosine deaminase: converting A to I to G Converting C to A, G, T	(55, 60, 61)
Search and replace	nCas9-RT	Conversion of eventually all possible genetic variants including mutation, insertion, deletion and repeat	(62)
**Transcription**			
Interference	dCas9-VP64 dCas9-KRAB	Regulating gene expression by recruiting transcriptional activator or repress to promoter or enhancer region	(63)
Epigenetic modification	dCas9-p300 dCas9-LSD1 dCas9-MQ1	Regulating gene expression through modification of Histone by methylation or acetylation.	(64–66)
**RNA**			
RNA targeting	Cas13a	Binding to target RNA and induce RNA degradation	(67)
Base editing	dCas13b-ADAR	Acting at RNA to convert A into C	(68)
RNA tracking	RCas9	Visualizing RNA transcripts in living cell	(69)
**Detection**			
DNA and RNA	Cas13a(C2c2)	Measuring DNA or RNA concentration	(70, 71)

Below, we review recent developments in the identification of genetic variants and the elucidation of possible molecular mechanisms underlying the functional defects observed in insulin secretion. We begin by providing examples of genes and *loci* associated with altered T2D risk. Finally, we review the CRISPR tools that may offer the potential to correct these variants in the human β cell.

## Variants Associated With Type 2 Diabetes

### MODY Genes

MODY is a clinically heterogeneous group of monogenic disorders characterized by β cell dysfunction and, in several cases, effects on other disease-relevant tissues including the kidney (74). Symptoms typically appear in adolescence or young adulthood (75, 76). MODY gene mutations are characterised by autosomal dominant inheritance and high penetrance. A total of 15 genes have been described to date, all involved in β cell function (**Table 1**). Mutations in the glucose-phosphorylating enzyme glucokinase (*GCK*), and the transcription factors *HNF1A* and *HNF4A* cause up to 80% of all MODY cases (74, 76, 77).

GCK

Glucokinase converts glucose into glucose-6-phosphate the flux generating step of glycolysis in the β cell and key control point for insulin secretion (78). Heterozygous loss-of-function mutations in the *GCK* gene induces a decrease of glucose phosphorylation into glucose-6-phosphate (G6P), which blocks the entry of G6P into the glycolytic pathway. Insulin secretion in response to glucose is reduced and this mechanism results in non-progressive fasting hyperglycaemia in patients (79–81).

*HNF1A* and *HNF4A*

Mutations in these two genes cause a progressive insulin secretory defect and hyperglycaemia (82–84). People with these forms of MODY respond well to low-dose sulphonylurea treatment, and sometimes insulin in later life (74). *HNF4A* and *HNF1A* regulate more than a dozen genes in human islets. Mutations in *HNF4A* cause downregulation of GLUT-2 (*SLC2A2*), aldolase B, and liver-type pyruvate kinase (L-PK) in β cells which lead to defective glucose sensing in pancreatic β cells (85). Additionally, *HNF1A* and *HNF4A* regulate each other, hence defects in either factor impacts the expression of the other (86). Heterozygous mutations in *HNF1A* downregulate the genes involved in glucose metabolism, including both *SLC2A2* and *PKLR* (87, 88). Ablation of *Hnf1a* in mice induces a reduction of β cell mass and β cell proliferation which together lead to a reduction of islet size (88). While ablation of *Hnf4a* in mice is embryonic lethal (89), expression of a dominant-negative form of *HNF1A* reduced the expression of genes involved in glycolytic and mitochondrial metabolism in these cells (90).

The other MODY gene variants associated with β cell dysfunction are rarer and the impacts more discrete (74, 91). Each type of mutation involves a specific type of MODY classified on the base of phenotypes, treatments, the extra-pancreatic features, the severity of hyperglycaemia and subsequently complications and prognosis (**Table 1**) (8, 91).

### GWAS-Identified Variants in Protein-Coding Regions

GWAS-identified variants associated with T2D risk include single nucleotide polymorphisms (SNP), deletions, insertions and short sequence repeats (6, 92). Although the majority of the variants reside in intergenic or intragenic regions, a few (less than 5%) are in protein-coding regions. As potential drug targets, these variant-containing genes have been subjected to investigation in β cells in recent years (5) using cellular and mouse knockout systems, as described in the examples below:

SLC30A8

This gene encodes ZnT8, a zinc transporter, which is highly and selectively expressed in pancreatic islet β and, to a lesser extent, α cells. ZnT8 transports zinc ions into insulin secretory granules and is thus implicated in insulin synthesis and secretion (93, 94). *SLC30A8* was first identified by GWAS in 2007 as hosting a variant affecting proinsulin processing and insulin secretion (26, 27, 39, 95). The common variant rs13266634 alters the amino acid sequence of the intracellular C-terminal domain of ZnT8 (Q325W) and has been the subject of extensive studies in the past decade. *Slc30A8* null mice exhibit reduced, unaltered or increased glucose-stimulated insulin secretion (GSIS) and glycemia (96–99), as reviewed earlier (94). However, recent studies focusing on rare genetic variants such as rs200185429 in which protective allele encodes a truncated ZnT8 protein (pArg138*) demonstrated that loss of ZnT8 improves insulin secretion and β cell function both *in vitro* and *in vivo* (39, 100–102). Thus, dose-dependent changes in appear to affect overall disease risk (94) likely reflecting the multiple roles of Zn^2+^ ions in the β cell.

PAM

*PAM* (Peptidylglycine α-amidating monooxygenase) encodes an α-amidase localized to the membrane of secretory granules which is involved in insulin granule packaging and release from β cells (103). Two GWAS-identified non-synonymous SNPs in the *PAM* gene, rs78408340 and rs35658696, affect T2D risk (28, 104, 105). Both SNPs are associated with a reduced insulinogenic index (a measure of glucose-stimulated insulin secretion), suggesting that their effects are mediated *via* altered β cell function (104, 105). The two SNPs fall in the coding region of the *PAM* gene and lead to the amino acid changes S539W and D563G, respectively. Thomsen et al. have demonstrated that PAM deficiency results in reduced insulin content and altered dynamics of insulin secretion in a human β-cell model and in primary islets from cadaveric donors (103). The risk alleles reduce overall PAM activity *via* defects in expression and catalytic function (103, 106).

### GWAS-Identified Genetic Variants at Non-Coding Genomic Locations

Whilst there are exceptions, such as those described above, most T2D-associated genomic variants lie in intergenic or intragenic regions. The underlying mechanisms through which these variants affect β cell function remain largely unknown though can involve altered transcription factor binding and thus modified expression of downstream target gene(s) (5). Examples of these and others are described below:

ADCY5

*ADCY5* encodes adenylate cyclase 5, a Ca^2+^-inhibited type III adenylate cyclase, which catalyses the generation of cyclic AMP (cAMP) from ATP. The risk variant at rs11708067 in intron 3 of the *ADCY5* gene is associated with elevated fasting glucose and implicated in defective proinsulin conversion to insulin (107–109). *ADCY5* mRNA expression in islets is lowered by the possession of risk alleles. Our own data showed that *ADCY5* silencing impairs glucose-induced cAMP increases and blocks β-cell glucose metabolism and intracellular signalling (110).

TCF7L2

This gene encodes the Wnt signalling-associated transcription factor, T-cell factor 7-like 2 (also termed transcription factor 7-like 2). Possession of risk alleles is associated with reduced glucose and glucagon-like peptide 1 (GLP-1) -stimulated insulin secretion (111). Functional analysis in β cell lines demonstrated that lower *TCF7L2* expression reduces insulin gene expression and glucose-stimulated insulin secretion (GSIS) (112) but not KCl-induced insulin secretion (113) and lowered the expression of β cell genes regulating secretory granule fusion at the plasma membrane. In animal models, selective deletion of *Tcf7l2* in the β cell (113, 114) replicates key aspects of the altered glucose homeostasis in human carriers of *TCF7L2* risk alleles.

STARD10

STARD10 is a phospholipid transfer protein possessing a steroidogenic acute regulatory protein (StAR)-related lipid transfer (“StART”) domain that facilitates the transport of phosphatidylcholine and phosphatidylethanolamine between intracellular membranes (115). Functional GWAS (fGWAS) identified a set of credible variants in the intron 2 of *STARD10* gene on chromosome 11 associated with impaired GSIS and, paradoxically but characteristically, with decreased proinsulin:insulin ratios (indicating improved proinsulin conversion) (116). In animal models, β cell-selective deletion of *StarD10* gene in mice led to impaired glucose-stimulated Ca^2+^ dynamics and insulin secretion. Conversely, overexpression of *StarD10* in the adult β cell improved glucose tolerance in high fat-fed animals (116). These data recapitulate the pattern of improved proinsulin processing observed at the human GWAS signal. *STARD10* inactivation reduces GSIS both in mice (116) and in human EndoC-βH1 cells (117) and leads to profound changes in secretory granule structure in mouse beta cells (118). Solution of the 3D structure of STARD10 and direct binding assays revealed that STARD10 binds to and may transport inositol phospholipids, contributing to the failure of normal granule biogenesis in carriers of risk alleles (where *STARD10* expression is lowered) (116).

*C2CD4A* and *C2CD4B*

Another genetic locus, present on chromosome 15q, and identified by GWAS (119) is associated with proinsulin levels and T2D risk. The risk variant of the single nucleotide polymorphism rs7172432 impaired GSIS in a non-diabetic Danish population (120). This SNP, together with others, lies in a stretch of the intergenic region between the *C2CD4A* and *C2CD4B* genes, which are located close to *VPS13C*, encoding a lipid transport protein (121) and which may also contribute to disease risk (122). In a recent study by Acilli and colleagues (123), β cell-selective deletion of *C2cd4a* in mice phenocopied the metabolic abnormalities of human carriers of polymorphisms at this locus, resulting in impaired insulin secretion during glucose tolerance tests as well as hyperglycemic clamps (123). Global deletion of *C2cd4b* leads to highly sexually dimorphic effects on glucose metabolism in mice (124) with evidence in females for actions in both the anterior pituitary -to modulate the secretion of follicle-stimulating hormone - and in β cells. On the other hand, and in contrast to the findings of others (123), who used the *Ins2*-depedent rat insulin promoter (RIP) promoter which can lead to deletion of β cells and in a subset of hypothalamic neurones in the ventromedial hypothalamus (125), systemic *C2cd4a* ablation had no effects on glucose homeostasis in the later study (124).

### Enhancer Clusters: Key Regulatory Regions Mapped by Epigenetics and Chromatin Structure

Transcriptional misregulation is involved in the development of many diseases including cancer, ageing and diabetes (126–128). Distal regulatory elements, such as enhancers, play a major role in specifying cell-specific transcription patterns in both normal and diseased tissues. In the diabetes field, enhancer or enhancer clusters have been a focal point in recent genetic and epigenetic studies of β cells (41, 42, 129). T2D variants are significantly and specifically enriched in islet-specific enhancer regions (41, 42) consistent with the role of variants in the regulation of target gene expression. Mohlke’s group focused on a functional SNP, rs11708067, overlapping with an enhancer in the *ADCY5* gene. These authors found that rs11708067 exhibits allelic differences in transcriptional activity and that deletion of this SNP-containing enhancer from rat INS1 (832/13) cells reduced the expression of *ADCY5* gene as well as insulin secretion (130). This work established the possible role of a non-protein coding SNP in the regulation of insulin secretion. Stitzel’s group carried out a detailed analysis of rs7163757 at the *C2CD4A/B* locus (131). Located within an enhancer cluster region, the risk variant of rs7163757 displayed higher transcriptional activity suggesting an increased expression level of *C2CD4A* and *C2CD4B* in diabetic β cells. Furthermore, the transcription factor nuclear factor of activated T-cells (NF-AT) was identified as a key factor to alter the transcriptional activity brought about by the risk variant and the increase in enhancer activity (131).

### Coregulation of Downstream Genes by Enhancer Clusters

Identified variants associated with diabetes risk, and their associated enhancer cluster, are often located far away in linear distance in the genome but physically interact with their downstream gene(s) through chromatin looping. To understand the spatial chromatin organization of human islets, chromosome conformation capture (3C) and related techniques such as (Hi-C) and promoter-Hi-C have been used to map 3D chromatin structure and to understand the interactions between enhancers and promoters (48, 129, 132–134). Through these approaches, it has become clear that enhancers, more specifically enhancer clusters, interact with multiple gene promoters through chromatin looping and simultaneously regulate multiple genes (47, 48). Causal variants are likely to influence enhancer activity and in turn entrain changes in signalling pathways in which these genes lie.

The study of non-coding variants involved in diabetes risk is still in its infancy. Nevertheless, as proof of concept, Ferrer’s group have demonstrated that alteration of variants containing enhancer activity by CRISPR interference affects the expression of multiple genes in EndoC-βH1 cells (48). Our own work, focusing on an enhancer cluster at the *STARD10* locus, also showed that an enhancer cluster regulates not only *STARD10* but also *FCHSD2* through chromatin looping (117). Detailed analyses of how these and other variants affect chromatin structure, enhancer activity and gene expression are now warranted to elucidate the molecular mechanisms of disease pathways.

## Genome Editing: Tools to Explore and Correct Genetic Defects

A challenge in modern medicine is to identify and correct mutations that lead to disease. In the context of studies in T2D these changes are usually (though not exclusively) most relevant in the pancreatic β cell [and as such are unlikely to impact the risk of other diseases, though this may be the case where the site of action, and causal gene(s), have roles outside of the pancreas]. As described above, different variants may play different roles and may require different strategies to correct at the genome level (135, 136). Towards this goal, early research indicated that double-strand DNA breaks (DSB) generated by an endonuclease can dramatically stimulate homology-directed recombination (HDR) in eukaryotic cells (137, 138). These observations led to the hunt for programmable and efficient endonucleases, leading to the development of meganucleases (139, 140). As part of the first generation of genome editing tools, meganuclease has shown its precision and effectiveness in genome editing (141, 142). However, given the length of its recognition sites—usually 12–40 bp—its practical application to genome editing is limited.

### Pre-CRISPR Era

Targeted DNA regions need to be rendered accessible using molecular “scissors”, subsequently allowing the DNA repair machinery to insert a sequence of interest. Providing two such tools are zinc finger nuclease (ZFN) (143) and transcription activator-like effector nuclease (TALEN) (144, 145) (**Table 2**). Both are engineered DNA restriction enzymes made by fusing the DNA binding domain of each to a DNA cleavage domain. ZFN uses zinc finger protein repeats while TALEN uses the humanized bacterial transcription activator-like (TAL) effector to bind to target DNA. The DNA cleavage domain in both cases is a catalytically active *Fok*I restriction endonuclease which effectively cuts both strands to induce DNA double-strand break (DSB). The cell then uses two DNA repair systems to repair DSB: the imprecise non-homologous end-joining (NHEJ) mechanism, which often generates deletion or mutations (termed “indels”) at the DNA cutting site, or the inefficient but precise homology-directed repair (HDR) mechanism using either single or double strand donor DNA as a template. Both tools have been used for genome editing in research and clinical settings. For examples, ZFN has been used to insert an *OCT4-eGFP* fusion gene into the *OCT4* gene locus (146) and to correct a mutation in the human *PIG-A* gene in embryonic stem cells (ES) and induced pluripotent stem cell (iPSC) by HDR (147). ZFN has also been designed against an X-linked severe combined immune deficiency (SCID) mutation in the IL2Rγ gene (148).

Due to its more advanced DNA binding design, TALE can be engineered to bind to practically any desired DNA target, and thus has been used more widely than ZFN (145). In the diabetes field, TALEN has been used to inactivate the *gagra* and *gcgrb* genes in zebrafish (149), the Sulfonylurea Receptor 1 (*Sur1*) gene in the rat (150), and several transcription factors in human iPSC cells (151).

The disadvantages of both ZFN and TALEN-based strategies, however, lie in their complicated design. The *Fok*I endonuclease must dimerize at the DNA binding domain in order to cleave DNA (152) and thus requires a pair of ZFNs or TALENs to target non-palindromic DNA sites. It is therefore difficult, especially when using ZFN, to design the DNA binding domain. Constructing the required DNAs consequently requires a high degree of skill in terms of both computational design and in molecular cloning.

### CRISPR-Cas9: A Simple and Efficient Editing Tool to Generate Mutations or Corrections in Genomic DNA

Research into mechanisms of bacterial immunity identified an effective DNA editing system termed CRISPR (see above) based on an RNA-guided endonuclease directed against the foreign pathogen (72, 73). Engineered CRISPR systems contain two components: a  single strand guide RNA (gRNA or sgRNA) and a CRISPR-associated endonuclease (Cas). The gRNA is a short synthetic RNA composed of a scaffold sequence necessary for Cas-binding and a user-defined 20 nucleotide spacer that defines the genomic target to be modified (**Figure 1** and **Table 2**). The target is present immediately adjacent to a Protospacer Adjacent Motif (PAM) (153). Thus, the genomic target of the Cas protein is determined by the gRNA and only restricted by the PAM sequence. Cas is an endonuclease which induces a double strand DNA break. Various humanized Cas proteins, including the commonly used SpCas9 from *Streptococcus pyogenes*, have been generated and diversified to suit different purposes (153, 154). In comparison with both ZFN and TALEN, CRISPR provides far simpler design and DNA construction strategies, with compatible DNA cutting efficiency (**Table 2**) (155).

#### Cas9

Due to its simplicity and adaptability, CRISPR has rapidly become the most popular genome editing tool available for the mammalian genome (50, 63). Because NHEJ DNA repair often introduces unwanted indels at the Cas9 cutting site, CRISPR has been used to knock-out genes by introducing frameshift mutations, resulting in protein depletion (156, 157). In the diabetes field, CRISPR has also been adopted to study several genes in β cell lines and in human ES-derived β cells (21, 151, 158, 159) as well as in animals (160, 161).

The insertion of precise genetic modifications by genome editing tools is, however, limited by the relatively low efficiency of HDR compared with the higher efficiency of the NHEJ pathway. For this reason, correction of genetic mutations such as those associated with MODY has met with limited success up to now. NEHJ-mediated DNA repair after Cas9 cutting has been shown to be non-random but with a pattern of indel formation dependent on PAM sequence (162, 163). Hence, it is possible, though the chance of success is low, to achieve precise DNA modification through the NEHJ pathway. One successful example is the restoration of FANCA gene expression in haematopoietic stem cells (164). HDR efficiency is generally low (less than 2%) but, with CRISPR technology, it can be improved to 10-40%, depending on the target region (165, 166). Several attempts have been made to improve HDR efficiency by incorporating silent CRISPR-Cas-blocking mutations (167), suppressing NHEJ key molecules such as KU70, KU80, or DNA ligase IV (168, 169), modification of RAD18 (165), providing asymmetric donor DNA (170) and applying chemicals such as scr7 (169).

In addition to gene knock-out and HDR repair, genome-wide pooled CRISPR-Cas9 libraries have been used to systematically delete genes responsible for diverse phenotypes. Recent studies have shown that such loss-of-function screens using libraries comprising tens of thousands of sgRNAs can be used to identify genes involved in tumour growth and metastasis (171). In the diabetes field, similar approaches have also been used recently to identify key insulin gene regulators (172) and the genes involving in auto-immune killing of β cell transplants (173). Screens based on transcriptional interference (CRISPRi) and activation (CRISPRa) have also harnessed Cas9-based technologies for use in genome-wide studies (59, 174). In addition, recent improvements in lentiviral library generation and propagation, as well as large-scale DNA and RNA synthesis, have allowed CRISPR-Cas9 technology to be exploited across multiple model platforms (59, 175–178).

#### nCas9

The CRISPR-Cas9 system can tolerate certain mismatches to the DNA target since the required gRNAs are short. A disadvantage, however, is that this can promote undesired off target mutagenesis (53). To overcome this problem, the Cas9 enzyme has been modified in its catalytic domain (D10A) (nCas9) which allows the enzyme to nick single strand DNA rather than double strand breaks (DSB) (**Figure 1** and **Table 2**). Because individual nicking is repaired with high fidelity, simultaneous nicking *via* appropriately offset guide RNAs is required for DNA double-strand breaks and extends the number of sites specifically recognized for target cleavage. This approach reduces off-target mutagenesis 50–1000-fold (53, 179). Furthermore, to improve HDR efficiency, nCas9 has been fused with RAD51 to insert disease-associated point mutations (180).

#### dCas9

Taking advantage of specific gRNA binding to target DNA sequences, CRISPR technology has been modified further to expand its applications in multiple ways (**Figure 1** and **Table 2**) (154, 181). Thus, in addition to its ability to cut double strand DNA or nick single strand DNA (nCas9), Cas9 has been modified with lowered endonuclease activity (D10A and 840) which allows the enzyme to bind to the target DNA without cleavage. The catalytically-dead Cas9 (dCas9) can be further engineered to fuse with many tail proteins for a range of applications: 1. Visualization of a genomic locus: dCas9 is fused with a fluorophore (i.e. eGFP) to enable sequence-specific visualization of DNA and dynamic imaging of chromatin (182, 183). To further improve this technique, several tag proteins such as Suntag (54), CASFISH (184), CRISPRainbow (55), and CRISPR-sirius (56) can be fused to dCas9; 2. Transcriptional regulation. dCas9 is fused with either a transcriptional activator (e.g. VP64) (185–187) or repressor (e.g. CREB) (182, 188). Once recruited by gRNA to the DNA target site, the activator or repressor brings in a transcriptional complex to enhance or repress gene expression. 3. Epigenetic regulation. dCas9 is fused with acetyltransferases (64) or demethylases (65) to engineer epigenetic changes in the genome. 4. Purification of genome regions. dCas9 is fused with epitope tag(s) such as FLAG or Biotin to facilitate the purification of the molecules associated with a genomic region of interest *in vivo* (57, 58, 189).

#### Improved Cas Proteins

Although widely used, CRISPR-cas9 is far from perfect as a genome editing tool. The widely-used *Sp*Cas9 requires an NGG PAM sequence for target recognition, thereby restricting the targetable genomic *loci*. The Cas9 protein is large and therefore difficult to propagate in a suitable viral vector. To overcome these limitations, several laboratories have further improved the usage of *Sp*Ca9 or identified alternative Cas proteins in the Cas family (**Figure 1**). For example, a version of Cas9 with high fidelity (Cas9-HF1) has been developed to reduce off-target effects (190). An engineered *Sp*Cas9 variant (i.e. SpCas9-NG) has been generated to recognize an alternative PAM sequence (191, 192). Some of the Cas9 homologs in the Cas family recognize different PAM binding sites relative to SpCas9, thereby offering alternative DNA targeting capabilities (193). For example, Cas12b has been rationally modified to facilitate robust genome editing and to exhibit greater specificity compared to Cas9 (194). A particularly exciting discovery has been the discovery of Cpf1/Cas12a, a DNA endonuclease of smaller size relative to SpCas9 (51, 195). Cpf1 can process its own CRISPR RNA (crRNA) and can be used to simplify multiplexed genome editing. Using a single customized CRISPR array, it has been reported that up to four genes can be edited simultaneously by CRISPR-Cpf1 (195). To improve the efficacy of CRISPR editing, an alternative strategy targeting CRISPR RNA (crRNA) has also been developed. Structure-guided chemical modification of crRNA by, e.g. a 2’ O-methyl (2’ OMe) sugar modification, has be used to protect nuclease digestion and stabilize the crRNA/Cas protein complex in cells while maintaining or enhancing genome editing activity (196–199).

#### RNA Editing

Further expansion of CRISPR-Cas9 tools led to the discovery of CRISPR on RNA targets. Cas13a, previously known as C2c2, is programmed to cleave a single-stranded RNA target carrying complementary protospacer in bacteria (67, 200) or in mammalian cells (67) (**Figure 1** and **Table 2**). Efficiency of silencing is comparable to RNA interference (RNAi) but offers improved specificity (67). Like dCas9, catalytically-dead Cas13a (dCas13a) has also been leveraged for programmable tracking of RNA transcripts in live cells (67, 201). In addition, Cas9 has also been modified for transcript tracking by RNA imaging (69). The latest addition of RNA editing comes from Type VI of CRISPR families termed CasRx (202).

CRISPR technology has also been developed as a diagnostic tool to detect DNA or RNA from biological samples. Examples include SHERLOCK (based on Cas13a) (69) and DETECTR (based on Cas12a) (203).

#### Limitations of CRISPR-Cas9

CRISPR provides a simple and easy tool not only for *in vitro* use but potentially also for *in vivo* genome editing. However, there are limitations and downsides to this approach. First, and despite considerable improvements in the technology, the risk of the off-target effect remains and must be considered carefully. Second, DSB may lead to wide-ranging deletions or recombination events involving the on-target site (204). Third, in cycling cells, DNA double strand breaks caused by Cas9 cleavage may trigger a P53 response leading to apoptosis and enrichment for potentially oncogenic P53-deficient cells (205, 206). Fourth, subjects may generate antibodies to Cas9, potentially limiting gene therapies (207, 208).

### Base Editing: Conversion of a Single Nucleotide

Most variants of interest in the diabetes field are single base pair changes (see above). These present a significant challenge for CRISPR-Cas9 genome editing since, until recently, low efficiency HDR was the only way these could be introduced. To overcome this problem, Liu’s group have used various deaminases to convert a single nucleic acid into another (60). In this way, a single nuclear variant (SNV) can be converted into another nucleotide (209) (**Figure 1** and **Table 1**).

#### Cytidine Deaminase

Cytidine deaminase catalyzes the conversion of cytosine into uracil (210, 211). The first cytidine base editor to be described was composed of dCas9 and the human apolipoprotein B mRNA editing enzyme (APOBEC) (60, 209, 212, 213). In the context of base editing, APOBEC deaminase is guided by dCas9 protein to the target DNA to convert the targeted C into U. The conversion results in a mismatch, U-G, which can be repaired by cellular mechanisms into U-A base-pair and eventually T-A base-pair (214) A second generation of cytidine deaminase-based DNA base editors was developed (BE2) using a chimeric protein of dCas9, APOBEC deaminase in addition to an uracil glycosylase (UGI) (60, 215). UGI achieves an error-free repair, which increases significantly the efficiency of base editing (216).

Apart from the human APOBEC protein, other types of cytidine deaminase have been coupled with either dCas9 or nCas9 (nickase) to introduce single point mutations. Activation-induced cytidine deaminase (AID) recruits the cytidine deaminase pmCDA1 which induce switch recombination or hypermutation in immunoglobulin produced by the human plasma cells in order to help the immune system neutralizing a larger number of pathogens through mutations in the fragment antigen-binding variable region (Fab). BE3 is based on the fusion of three proteins: dCas9, pmCDA1, and UGI (214, 217). This system, in common with earlier versions, is limited by the creation of indels. BE4, using two UGIs instead of one, appears to be more efficient than BE3.

Other CRISPR endonucleases such as Cpf1 can also be fused with deaminase. The chimeric deaminase coupling Cpf1, APOBEC and UGI (dLbCpf1-BE0) further extends the base-editing capacity to target sequences which cannot be reached by the Cas9 machinery (214, 218).

#### Adenosine Deaminase

Adenine can be deaminated to become inosine (219). Eukaryote polymerases subsequently base-pair inosine to guanosine, converting A into G (209, 214).

The first-generation adenine base editors were developed in bacteria. *E. coli* resistant to chloramphenicol acquire an adenine editing domain of edTAd-cas9 after antibiotic selective pressure (214). Adenosine deaminase based base editors (ABE) are able to deaminate adenine on single-stranded DNA and convert adenine into inosine (61, 209, 214).

#### Cytidine to the Three Other Nucleic Acids

The deamination process can also be used to convert cytosine and guanine to a diverse library of point mutations localized to a targeted region of the genome. Two new technologies, Targeted AID-mediated Mutagenesis (TAM) (220) and CRISPR-X (221), mimic the somatic hypermutation process. This process is generally useful during antibody affinity maturation, to generate localized sequence diversification. The TAM system is composed of dCas9 and activation-induced cytidine deaminase (AID) (214) and the CRISPR-X system, is composed of a dCas9 which recruits a hyperactive variant of the AID enzyme AID* (214, 221).

#### RNA Base Editing

ADAR proteins are adenosine deaminases that act on RNAs by converting adenosine to inosine. Inosine is read as guanine by the translational machinery, thereby base pairing with cytosine (214, 222). ADAR2 is an RNA-guided editor system which uses a catalytically-dead Cas13b (dCas13b) to localize an ADAR protein and convert A-I in a target double-stranded RNA causing an A-C mismatch (68, 222).

#### Limitation

Cytosine DNA base editors (CBE) have specificity limitations because of the uracil N-glycosylase activity (UNG). UNG is involved in unanticipated C-to-non-T edits (60, 209, 223). Therefore, cytosine base editors fused with one or more UNG inhibitors (UGI) show a significant increase in their editing specificity. In addition, some point mutations can allow overexpression of UGI *in trans* which can further improve UGI activity and thereby the editing product purity (60). The cytosine base editor also exhibits indel formation which can be reduced by fusing the bacteriophage Mu-derived Gam (Mu-GAM) protein to CBE (224).

In the case of a target sequence exhibiting several C or A bases, conversion of bases in addition to the target base can occur (“bystander editing”). Base editing systems with wide editing windows are more likely to suffer from this problem (225). Some specific mutations in the APOBEC1 domain have been shown to reduce deamination activity and lower bystander editing.

### Search and Replace: A New Tool With a Simpler and Versatile Way of Genome Editing

Both CRISPR-Cas9 and base editing provide easy and rapid gene editing approaches, but they lack precision and often cause unwanted on- and off-target effects. In some cases, double strand DNA breaks can produce large deletions in nearby genome elements (204). Addressing some of these issues a new editing tool, termed prime editing or search and replace, has been developed recently by David Liu’s group (62) (**Figure 1** and **Table 2**). Here, a catalytically-impaired Cas9 (nCas9) fused to an engineered reverse transcriptase (nCas9-RT) is programmed with a prime editing guide RNA (pegRNA) that both specify the target site and encode the desired edit. Because this approach uses nickase (nCas9), it offers much lower off-target editing than Cas9 nuclease, and thus generates fewer by-products. This strategy also offers efficiency and product purity advantages over HDR replacement, and complementary strengths and weaknesses compared to base editing.

### *In Vitro* and *In Vivo* β Cell Models for Studying Genetic Variants

In order to understand the pathogenic role of diabetes-associated genetic variants, tractable β cell models are essential. Mouse models, either transgenic or knock-out, are valuable for examining the roles of single genes, but their use is more limited in studies of intergenic regions given more substantial inter-species (mouse versus human) differences in these regions. As sources of human β cells, there are currently three possibilities. Firstly, primary islets isolated from organ donors: This source is, however, limited in terms of the availability and quality of islets (226). Secondly, clonal human β cells. Immortalized human EndoC-βH1 cells were developed in recent years after infection of foetal islets with large T antigen and further inoculation of islets in immunocompromised mice (227). Later generation EndoC-βH2 (228) and EndoC-βH3 (229) cell lines were subsequently established with more advanced features including regulated deletion of the immortalizing gene. The limitation of these cell lines, however, is their extremely slow growth rate which hampers their use. Given this slow growth rate—and the fact that these lines poorly tolerate expansion from a single cell—it is virtually impossible to modify them by HDR *via* CRISPR editing. A third possibility are therefore islet-like cells differentiated from human embryonic stem cells (hESC) or patient-derived induced pluripotent stem cells (iPSC). In light of the limitations of the above cellular models, laboratories are now focusing on hESC or iPSC in studies of gene function throughout β cell development by differentiating hESC/iPSC cells into mature β cells (230, 231). Such directed differentiation protocols have recently been improved (21, 159).

## Application of Genome Editing to Disease-Relevant Genetic *Loci* In β Cells

*MODY*. Animal models have been generated using CRISPR-Cas9 technology to study features of *HNF4A* (232), as well as a *GCK* mutant rabbit model exhibiting many features of HNF4A (233) and *INS* mutant piglets which are insulin-deficient (161). Similarly, a CRISPR-Cas9 nuclease was used to create the MODY gene reporter through homologous recombination, such as *PDX1-eGFP* reporter (234). In the latter study, a CRISPR-on system was also fused as a transcriptional activator (dCas9-VP160) to activate the transcription of endogenous human *INS* gene in human stem cells (235).

Patient-derived human induced pluripotent stem cells iPSCs from people with diabetes can be used to model disease *in vitro*, and many labs have made huge efforts to generate iPSC lines from people with MODY (236). Although most of the resulting lines have not been fully characterized (given cost limitations and the challenges associated with β cell differentiation), interesting data have been obtained. For example, an iPSC line carrying a heterozygous mutation in HNF1B^S148L/+^ exhibited compensatory up-regulation of several key endocrine pancreas-enriched transcription factors including PDX1 during β-cell differentiation (237). Other *HNF1B* mutant lines were also generated from people carrying *HNF1B* mutations (236–238). *HNF1A* mutant lines were established from patients with different variants (239) including *HNF1A*^S142F^ (240) and *HNF1A*^P291fsinsC^ (236). Similarly, a *GCK* mutant line carrying GCK^V62A^ has been established (236). *HNF1A* and *CEL* lines have also been generated from patient samples (236).

CRISPR technology has been used recently to correct point mutations in patient-derived iPSCs to target diabetes-related gene defects. To date, the most efficient method used in iPSC is CRISPR/Cas9-based homology-directed repair (HDR). Here, a Cas9-mediated cut is generated adjacent to the site of interest. A homologous donor template with the intended nucleotide change containing silent mutations in the gRNA sequence (167) can then be recombined by HDR. This approach has been used successfully to investigate *STAT3* and *GATA6* mutant iPSC lines generated by reprogramming patient cells expressing a heterozygous mutation (241, 242). Mutations in both genes were corrected with CRISPR/Cas9 and completely reversed the disease phenotype.

### GWAS-Identified Genes

Following the successful identification of genetic *loci* by GWAS, several candidate genes within or surrounding genetic *loci* which are thought to play roles in β cell function, in particular, in proinsulin processing and secretion, have been examined in mechanistic studies. Gene editing tools have quickly replaced techniques such as shRNA-based silencing and HDR-mediated deletion to become a mainstream technique in studies of gene function. For example, the critical β cell-enriched *NEUROD1* and *SLC30A8* genes were deleted in EndoC-βH1 cells using these approaches in recent studies (243). Similarly, pancreatic duodenum homeobox-1 (PDX1), an important regulator of the *INS* gene, was also mutated by CRISPR-Cas9 resulting in a line with defective glucose-induced Ca^2+^ influx and insulin secretion (244). Our laboratory has inactivated the type 2 diabetes-related *STARD10* and *FCHSD2* genes in EndoC-βH1 cells using a lentiviral approach and demonstrated effects on insulin secretion (and see above) (117). Furthermore, Fang et al. used CRISPR screening technology and identified several genes involved in insulin regulation in mouse MIN6 cells (172).

Gene editing in hESC/iPSC cells has also been documented. The *NEUROG3* gene, a transcription factor essential for the development of pancreatic endocrine cells in mice, was knocked-out by CRISPR-Cas9 in hESC cells and confirmed there was no endocrine cells formed from pancreatic progenitors (245). Chen’s lab used CRISPR-Cas9 to knock out three β cell-specific transcription factor genes in hESC cells and proved the usefulness of this hESC cell platform (158). Similar studies were reported by Huangfu and colleagues who used either TALEN or CRISPR-Cas9 to systematically delete several β cell transcription factors, demonstrating roles in human β cell development and function (21, 151).

### Enhancers and Genetic Variants

Genetic enhancer elements, critical determinants of cellular identity, are usually identified based on chromatin marks and gain-of-function potential, though only loss-of-function studies can demonstrate their requirement in the native genomic context. Various CRISPR technologies have been applied to identify potential enhancer regions (177), the critical transcriptional factor binding site for gene expression (246), long-range gene regulation in normal and malignant cells (247–249).

The application of CRISPR to studies of enhancers in β cells is still at an early stage. Malkon’s laboratory (130) have deleted a variant-containing enhancer region within the *ADCY5* gene in INS1 cells and demonstrated reduced *ADCY5* gene expression. Also using CRISPR-Cas9, we deleted an active enhancer within an enhancer cluster in the *STARD10* locus in EndoC-βH1 cells which reduced the expression of both the *STARD10* and *FCHSD2* genes (117). Moreover, CRISPR interference techniques (CRISPRi and CRISPRa) have been used to modulate the transcriptional activities of several enhancer regions in GWAS-identified genetic *loci* to demonstrate that altered enhancer activity impacts the expression of multiple genes within enhancer hub (48). A similar attempt was also carried out on enhancer activity by CRISPRa to increase endogenous human *INS* gene expression (250).

Attempts to correct genetic variants *via* HDR have also been reported. Mutations in the *INS* gene affecting insulin (M1I) or proinsulin (C96R, C109Y) were corrected by HDR *via* CRISPR-Cas9 editing to restore insulin production in differentiated iPSC cells that mimicked neonatal diabetes (251, 252). Likewise, Shi et al. converted a patient-specific mutation in *GATA6* gene and showed that the mutation involved (GATA6^R456C^) has a similar effect to *GATA6* knockout (21). Most recently, correction of a variant in the Wolfram syndrome 1 (*WFS1*) gene by CRISPR-mediated HDR improved insulin secretion in iPSC-differentiated β-like cells (253). Studies on GWAS identified genetic variants especially those in the intragenic and intergenic regions have not been reported. Given their functional importance on chromatin structure, enhancer activity and gene regulation, it is predicted that variant conversion in human β cells will attract huge interests in near future studies.

### Genome Editing in Animal Models

Over the past three decades, gene knockout in animals, especially in mice, has provided invaluable information of gene function (254). The traditional way of gene deletion carried out in embryonic stem cells (ES) is through homology-directed recombination to delete a piece of genomic DNA, such as an exon, to achieve systemic (whole body) knockout. Tissue or cell-selective gene knockout can be achieved by using the “Cre-loxP” system (255, 256). CRISPR-Cas9 mediated gene knockout, as a relatively new approach, provides a much simpler and effective way to achieve gene knockout *in vivo*, though the generation of LoxP sites flanking a gene or exon of interest is more challenging. In brief, the gRNA is synthesized, or *in vitro* transcribed, and then complexed with Cas9 protein to form a ribonucleoprotein (*RNP*) complex. This complex can be directly injected into fertilized mouse zygote (156) or electroporated *in situ* into the oviduct of a pregnant female mouse (257, 258). The complex exerts its effect at target DNA by generating indel (with one gRNA) or deletion (with two gRNAs) mutations or DNA replacement through HDR with a donor template (156, 156, 259, 260).

Conditional knockout (cKO) mice are also an extremely valuable tool in biomedical research because they enable detailed analyses of gene function in a tissue- and/or temporal-specific fashion. However, the conventional method for generating cKO mice is time-consuming and labour intensive, and involves making a large gene-targeting construct, transfecting and screening many embryonic stem (ES) cell clones, injecting positive ES clones into blastocysts to produce chimeric mice, and breeding the chimaeras to transmit the targeted gene through the germline. This procedure can be substantially simplified by providing a CRISPR ribonucleoprotein RNP and single strand edDNA (ssDNA) donor which carries desired changes such as insertion of loxP site (255, 259–265). Using CRISPR-Cas9, leptin and leptin receptor knockout mice have been established as tools in diabetes and obesity research (160, 255, 256). Knock-in mouse models have also been established *via* HDR to achieve cell-specific deletion of the gene (266).

### Genome Editing: Clinical Application in Diabetes

**Figure 2:**
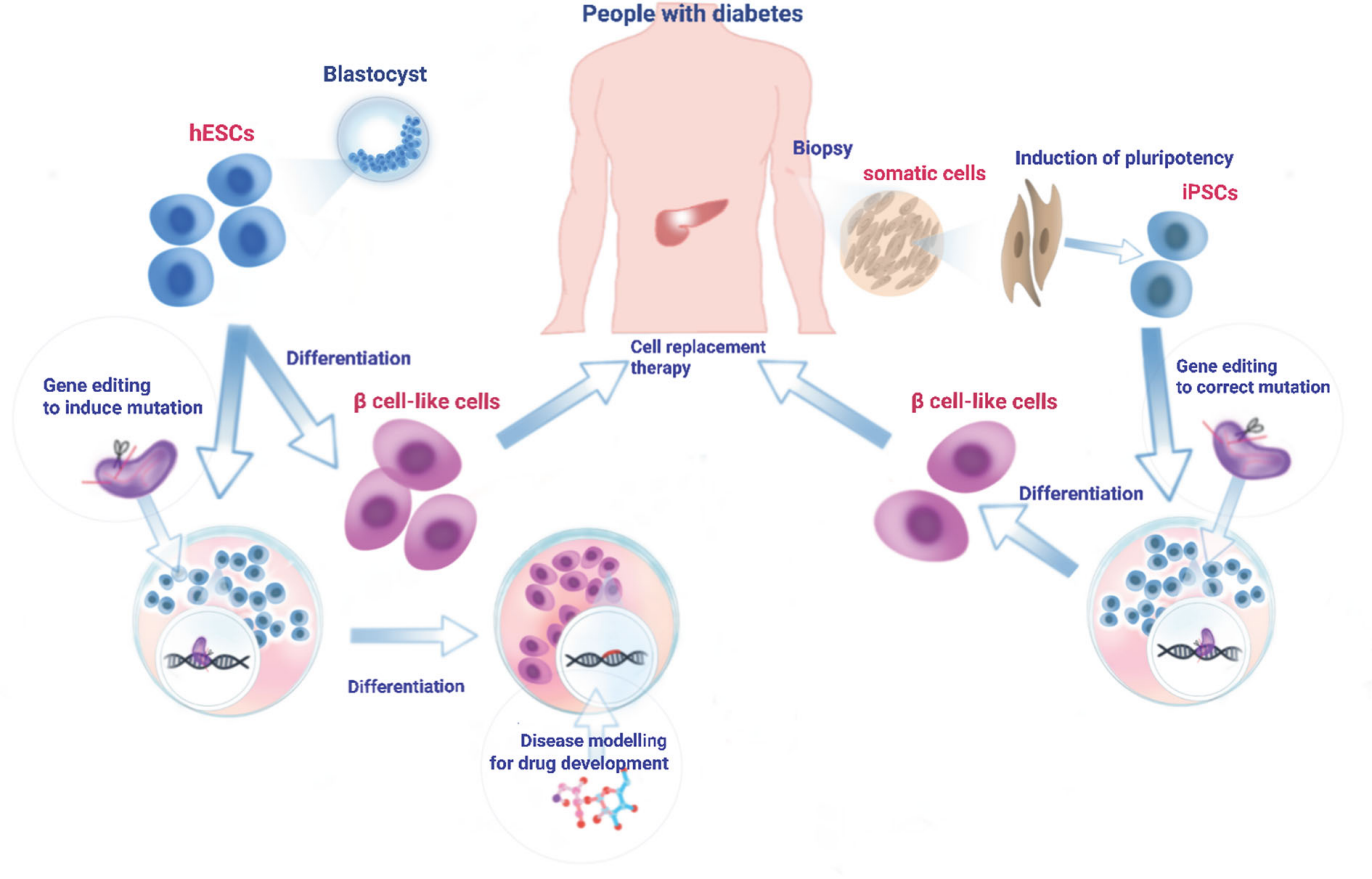
Generation, differentiation, and transplantation of iPSC cells into diabetic patients. A biopsy (skin fibroblasts, cord blood, or peripheral blood cells) is obtained from diabetes patient and cultured in the laboratory. Cultured cells are reprogrammed into iPSC cells using Yamanaka factors (271). To correct genetic mutation(s) or convert risk variant(s) into protective variant(s), iPSC can be edited at the genome level using one of the genome-editing tools. Engineered iPSC cells are then differentiated into β cell-like cells through a stepwise differentiation protocol by exposing the cells to specific growth factors/cytokines and signalling proteins. Alternatively, hESCs derived from healthy individuals can be cultured in the laboratory and driven to differentiate towards mature β cells. Those *in vitro* generated β cell-like cells which are free of mutation(s) can then be transplanted into patients to reverse diabetes. In addition, hESCs can be genome-edited in reverse to create disease-related mutation(s) and then subjected to directed β cell differentiation. Such mutation-bearing β cell-like cells can be used for the molecular mechanistic studies of disease as well as drug screening to identify therapeutic reagents.

An important goal in genetic research is to identify the genetic defects underlying disease pathogenesis and introduce corrections to restore normal gene function. In this respect, CRISPR-based technologies hold enormous potential in a therapeutic setting, offering an approach to permanently correct disease-causing mutations. The delivery of genome editing tools to the target cells can be broadly categorized into *in vivo* and *ex vivo* approaches. Both approaches have been extensively practised in the broader gene and cell therapy field and have achieved some clinical success (267). In recent years, such delivery strategies have also been extended to CRISPR-based therapeutics (267, 268). *In vivo* delivery aims to introduce genome editing reagents into patients systemically or locally to directly manipulate cells in the body (269). In *ex vivo* delivery, genome editing reagents are introduced into isolated human cells to achieve the desired genetic modification. After expansion, the genetically-modified cells are infused into patients to confer a therapeutic effect (270) (**Figure 2**).

*In vivo* delivery of CRISPR editing tools into pancreatic β cells in people with diabetes is likely to face enormous challenges for two main reasons: 1. β cells are postmitotic, thus disfavouring HDR-mediated CRISPR editing. 2. Selective targeting to these cells will be required, likely involving cell type-tropic viruses (272), raising evident concerns over off-target effects and toxicity. Hence, the most likely and feasible way of CRISPR editing has to be an *ex vivo* system where β cells can first be engineered by CRISPR editing and then transplanted into patients (**Figure 2**).

Ex Vivo

A major problem for cell-based treatment for diabetes patients is the lack of a suitable β cell source. hESC and iPSC cells provide potential means to produce sufficient amounts of high quality β cells for transplantation. Protocols for hESC/iPSC differentiation towards mature β cells were readily established in several laboratories (158, 230, 231, 273, 274). Importantly, these *in vitro* differentiated β like cells have the same physiological functions as mature β cells, i.e. producing and secreting insulin in response to various stimuli including glucose after transplantation in an immunocompromised mouse model (230, 231). However, the use of iPSC is controversial and there are some concerns over genetic and epigenetic variations in iPSCs which might affect cell function after differentiation (275).

Manipulation of hESC/iPSC cells *via* CRISPR-Cas9 technology provides a platform for the correction of genomic mutations not only in diabetes but in other disease fields as well (276–279). Through CRISPR-mediated HDR and base editing, it is possible to correct the vast majority of genetic variants, if not all. Conversion of GWAS-identified non-coding variants has not been conducted/documented in the diabetes field, but it seems inevitable that such work will be carried out in the near future given its importance in basic research and potential clinical application. Variants identified by GWAS are often clustered in the genome (134). Although an individual variant may change transcription factor binding on its own, neighbouring risk variants might cooperate to change the transcriptional landscape of local chromatin and thus the activity of the enhancer cluster leading to changes in the expression of multiple genes whose aggregate effect is to impair β cell function. Hence, multiplex genome-editing needs to be carried out to convert multiple risk variants into protective (non-risk) variants in hESC or iPSC cells. In this case, the off-target effects brought by multiplex gRNAs may have a large impact on the rest of the genome and raise major concerns.

In view of the above, genome editing tools need to be carefully selected. The newly developed nCas9-RT holds great potential: 1. The nCas9 nicks the DNA rather than induces DSB and therefore avoids indel formation at the cutting site; 2. The use of pegRNA, which is a combination of gRNA, reverse transcription template and primer-binding sites, increases the specificity of target DNA binding hence reduces off-targets (62); 3. While multiplex pegRNAs could target various variants including SNPs, deletions or insertions without separating DNA donors as templates, it is possible the nCas9-RT will be able to convert all variants at once. This new technique, however, is still in early development, and its editing efficiency and side-effects remain to be seen.

## Future Prospectives

Recent technological developments around CRISPR-Cas9 and its derivative technologies, combined with advances in human cellular models, should accelerate our understanding of the interplay between diabetes risk-associated genetic variants and their functional roles in disease pathogenesis. These approaches may also find use in clinical applications and in drug screens (**Figure 2**), enhancing the development of precision medicines for personalized treatment.

## Author Contributions

MH and IC wrote the first draft of the manuscript. SM provided comments and suggestions especially on MODY genes. GR devised the structure of the manuscript and revised the draft. All authors contributed to the article and approved the submitted version.

## Funding

GR was supported by Wellcome Trust Senior Investigator (WT098424AIA) and Investigator (212625/Z/18/Z) Awards, MRC Programme grants (MR/R022259/1, MR/J0003042/1, MR/L020149/1) and Experimental Challenge Grant (DIVA, MR/L02036X/1), MRC (MR/N00275X/1), Diabetes UK (BDA/11/0004210, BDA/15/0005275, BDA 16/0005485) and Imperial Confidence in Concept (ICiC) grants, and a Royal Society Wolfson Research Merit Award. This project has received funding from the European Union’s Horizon 2020 research and innovation programme *via* the Innovative Medicines Initiative 2 Joint Undertaking under grant agreement No 115881 (RHAPSODY). This Joint Undertaking receives support from the European Union’s Horizon 2020 research and innovation programme and EFPIA.

## Conflict of Interest

GR has received grant funding from Les Laboratoires Servier and is a consultant for Sun Pharmaceuticals.

The remaining authors declare that the research was conducted in the absence of any commercial or financial relationships that could be construed as a potential conflict of interest.

## Abbreviations

DSB, double-strand breaks; HDR, homology-directed DNA repair; T1D, T2D, type 1, type 2 diabetes.
